# Deciphering the Role of Innate Immune NF-ĸB Pathway in Pancreatic Cancer

**DOI:** 10.3390/cancers12092675

**Published:** 2020-09-19

**Authors:** Namrata Khurana, Paarth B. Dodhiawala, Ashenafi Bulle, Kian-Huat Lim

**Affiliations:** Division of Oncology, Department of Internal Medicine, Barnes-Jewish Hospital and The Alvin J. Siteman Comprehensive Cancer Center, Washington University School of Medicine, St. Louis, MO 63110, USA; nkhurana@wustl.edu (N.K.); dodhiawalap@wustl.edu (P.B.D.); ashenafibulle@wustl.edu (A.B.)

**Keywords:** NF-κB, pancreatic cancer, inflammation, IRAK4, TPL2, TAK1

## Abstract

**Simple Summary:**

Chronic inflammation is a major mechanism that underlies the aggressive nature and treatment resistance of pancreatic cancer. In many ways, the molecular mechanisms that drive chronic inflammation in pancreatic cancer are very similar to our body’s normal innate immune response to injury or invading microorganisms. Therefore, during cancer development, pancreatic cancer cells hijack the innate immune pathway to foster a chronically inflamed tumor environment that helps shield them from immune attack and therapeutics. While blocking the innate immune pathway is theoretically reasonable, untoward side effects must also be addressed. In this review, we comprehensively summarize the literature that describe the role of innate immune signaling in pancreatic cancer, emphasizing the specific role of this pathway in different cell types. We review the interaction of the innate immune pathway and cancer-driving signaling in pancreatic cancer and provide an updated overview of novel therapeutic opportunities against this mechanism.

**Abstract:**

Pancreatic ductal adenocarcinoma (PDAC) is one of the most lethal cancers with no effective treatment option. A predominant hallmark of PDAC is the intense fibro-inflammatory stroma which not only physically collapses vasculature but also functionally suppresses anti-tumor immunity. Constitutive and induced activation of the NF-κB transcription factors is a major mechanism that drives inflammation in PDAC. While targeting this pathway is widely supported as a promising therapeutic strategy, clinical success is elusive due to a lack of safe and effective anti-NF-κB pathway therapeutics. Furthermore, the cell type-specific contribution of this pathway, specifically in neoplastic cells, stromal fibroblasts, and immune cells, has not been critically appraised. In this article, we highlighted seminal and recent literature on molecular mechanisms that drive NF-κB activity in each of these major cell types in PDAC, focusing specifically on the innate immune Toll-like/IL-1 receptor pathway. We reviewed recent evidence on the signaling interplay between the NF-κB and oncogenic KRAS signaling pathways in PDAC cells and their collective contribution to cancer inflammation. Lastly, we reviewed clinical trials on agents that target the NF-κB pathway and novel therapeutic strategies that have been proposed in preclinical studies.

## 1. Introduction

Pancreatic ductal adenocarcinoma (PDAC) has recently emerged as the third leading cause of cancer-related death in the US and is projected to be the second by 2030 [1]. Due to a lack of early symptoms and effective screening strategies, only 10–15% of PDAC patients are diagnosed at an early stage that allows surgical resection. For these patients, adjuvant chemotherapies are routinely offered [2,3,4]. Yet, the majority of these patients succumb to disease relapse, indicating the strong resistance of PDAC cells to chemotherapy. For patients with inoperable or metastatic diseases, combination chemotherapies including FOLFIRINOX (cocktail of 5-FU, oxaliplatin, leucovorin and irinotecan) and gemcitabine/nab-paclitaxel are the mainstay treatment [5,6], but treatment response is neither universal nor durable. This dire scenario translates into an estimated 47,050 deaths, or ~82% of new 57,600 PDAC cases diagnosed in the US in 2020 [7]. The 5-year survival rate for all PDAC patients is currently at ~9%, the lowest among all major cancer types. Despite decades of intensive research from the academia and industry, newer treatment modalities including molecular-targeted and immunotherapies, which are part of standard treatments for other cancer types, remain largely unsuccessful in PDAC.

Several factors, both intrinsic and extrinsic, contribute to the aggressive behavior of PDAC. PDAC cells are intrinsically driven by powerful oncogenic mutations, including activating KRAS mutations, loss of TP53, and CDKN2A/B and SMAD4 tumor suppressor genes [8], which endow PDAC cells with superior capabilities to survive in adverse environments, withstand therapeutic attacks, and metastasize. Externally, the tumor microenvironment (TME) of PDAC is characterized by a thick, densely fibrotic (desmoplastic) matrix consisting of collagen, hyaluronan, and fibronectin, which can constitute up to 80–90% of the tumor bulk [9]. Studies over the past two decades have shown that the desmoplastic stroma not only limits vascularity and delivery of therapeutics but is also heavily infiltrated with suppressive immune cells that incapacitate anti-tumor T cells [10,11,12,13]. However, addition of stroma-depleting agents, especially sonic Hedgehog inhibitors or pegylated hyaluronidase, to chemotherapy failed to benefit patients in clinical trials [14,15,16,17]. Furthermore, mouse models suggest that depletion of stromal fibroblasts alone carries a risk of reverting PDAC cells to a progenitor-like and aggressive state that is more treatment-resistant [13,18]. Therefore, an in-depth understanding of the tumor-intrinsic and -extrinsic signaling pathways that contribute to desmoplasia is essential in devising effective therapeutic strategies.

## 2. Chronic Inflammation Drives Desmoplasia and Neoplastic Progression in PDAC

Chronic inflammation is the central mechanism that drives desmoplasia and neoplastic progression in PDAC [19]. The driving force of inflammation can originate from both neoplastic cells and external environmental stimuli. In genetically engineered mouse models (GEMMs), expression of oncogenic KRAS (such as KRAS^G12D^) and loss-of-function Trp53 mutants in pancreatic lineage cells (p48-Cre or PDX-Cre; Trp53^WT/R172H^ or Trp53^WT/flox^; LSL-KRAS^G12D^, generally termed KPC mice) results in the formation of highly desmoplastic PDAC [20,21,22], strongly suggesting that secreted factors or physical cues from the neoplastic PDAC cells are sufficient in driving desmoplasia. On the other hand, in the absence of Trp53 mutations, p48-Cre, or PDX-Cre; LSL-KRAS^G12D^ (or KC), mice have very low penetrance of developing PDAC [21]. However, the addition of external inflammatory stimuli, such as by treating mice with caerulein [23,24], cigarette smoking [25] or a high fat diet [26], can greatly accelerate the development of highly desmoplastic PDACs. These latter scenarios are distinctly reminiscent of human patients, where autoimmune pancreatitis, alcoholism, smoking, obesity, chronic biliary inflammation, and advanced age increase the lifetime risk of developing PDAC [27,28,29]. In addition, PDAC patients are characterized by significant cachexia even at an early stage of diagnosis, largely due to increased serum levels of pro-inflammatory cytokines interleukin (IL)-1α/β, IL-6, tumor necrosis factor (TNF)-α, and interferon (IFN)-γ [30]. Therefore, chronic inflammation is a core component in the pathophysiology of PDAC, from tumor initiation to progression to clinical manifestations.

## 3. NF-κB Pathway: A Major Driver of Inflammation in PDAC

Aberrant activation of the NF-κB family of transcription factors is perhaps the most common and dominant mechanism that drives chronic inflammation in human cancers. The NF-κB factors comprise of five different members: RELA (p65), RELB, c-REL, p50/p105, and p52/100 [31]. They are classified as NF-κB/Rel proteins as they all share a Rel homology domain (RHD) in the N-terminus, which is critical for homo- or hetero-dimerization and binding to κB cognate DNA elements in target genes. The activity of NF-κB is principally regulated by inhibitors of κB (IκBs) which mask the nuclear localization signals (NLS) of NF-κB, keeping them sequestered in an inactive latent complex in the cytoplasm [32]. There are canonical and non-canonical NF-κB pathways. In the canonical pathway, the IκB kinases (IKK) phosphorylate IκB upon receiving extracellular signals, such as cytokines, stress, free radicals, or radiation, resulting in the polyubiquitination and proteasomal degradation of IκB. This leads to the release of p65 and p50 which can translocate into the nucleus to transactivate κB-dependent genes [33]. The non-canonical pathway involves p100/RelB complexes which, at baseline, are inactive in the cytoplasm. Signaling through receptors, such as CD40 and the lymphotoxin β receptor (LTβR), activates the NF-κB-inducing kinase (NIK), which in turn activates IKKα, leading to phosphorylation of p100 at the C-terminal residues. This results in polyubiquitination and proteasomal processing of p100 to p52 which can translocate into the nucleus and complex with RELB to transactivate target genes [34]. In PDAC, the canonical pathway is the main driving mechanism of NF-κB activity.

### 3.1. The Role of NF-κB in PDAC Cells

Constitutive activation of NF-κB occurs in ~70% of PDAC samples [35,36], as seen by increased immunohistochemical staining of phosphorylated or nuclear RELA in neoplastic cells. Apart from inflammation, the NF-κB transcription factors control genes that contribute to various hallmarks of cancer, which include proliferation, evasion from apoptosis, enhanced angiogenesis, metastasis, and invasion [33,37,38]. Several review articles have been published delineating the pro-tumorigenic roles of NF-κB in PDAC, and these will not be described in detail here. Importantly, NF-κB activity can be further induced under stress conditions, including DNA damage, and is a major mechanism that confers resistance to chemotherapeutic agents, such as gemcitabine [39,40,41,42]. Mechanistically, NF-κB activation slows down the cell-cycle, thereby desensitizing PDAC cells to chemotherapy, inducing anti-apoptotic proteins that block the caspase activation, and inducing stemness [43,44].

### 3.2. The Role of NF-κB in CAFs

Cancer-associated fibroblasts (CAFs) play a major role in treatment resistance and progression of PDAC [45,46]. However, near depletion of CAFs paradoxically promotes the development of more aggressive and poorly differentiated PDAC [13,18]. It is now clear that PDAC CAFs consist of at least three different transcriptomic subtypes: inflammatory CAFs (iCAFs), myofibroblastic CAFs (myCAFs), and antigen-presenting CAFs (apCAFs) [47,48]. Robust phosphorylation of RELA was observed in a subset of PDAC CAFs and is critical for collagen deposition and secretion of inflammatory cytokines, including IL-6 and IL-1β [49], suggesting NF-κB to be the driving force in iCAFs. Importantly, the abundance of IL-1β staining in CAFs is associated with poor prognosis. Mechanistically, RELA activation in CAFs is driven by IL-1β secreted from CAFs, and surrounding PDAC cells and can be blocked by interleukin-1 receptor-associated kinase (IRAK)4 inhibition. PDAC cells injected into IRAK4-null mice or co-injected with IRAK4-silenced CAFs develop markedly smaller and less fibrotic tumors [49]. Notably, IRAK4 inhibitors markedly reduce tumor fibrosis and synergize with gemcitabine, leading to significantly better tumor control. These results provide a tractable strategy to selectively target iCAFs to improve therapeutic response. In addition, recent evidence has shown that pancreatic stellate cells (PSCs) secrete chemokine (C-X-C motif) ligand 2 (CXCL2) by engaging p50 to block CD8^+^ T cell infiltration in PDAC [50]. This further supports the rationale to target the NF-κB cascade in CAFs.

### 3.3. The Role of NF-κB in Immune Cells

The role of NF-κB factors in immune cells is extremely complicated, context dependent, and mostly studied using conditional knockout mouse models. The role of NF-κB in each immune subset in PDAC is largely unclear. In PDAC, certain subsets of myeloid-derived suppressor cells (MDSCs) and tolerogenic regulatory T (Treg) cells actively contribute to tumor progression and treatment resistance [51,52]. Granulocytic MDSCs (G-MDSCs) constitute 70–80%, or higher, whereas mononuclear MDSCs (M-MDSCs) constitute 20–30% of the total population of MDSCs. On the other hand, anti-tumor CD4^+^ and CD8^+^ T cells are either scarce or dysfunctional. The crosstalk of MDSCs with immune cells, such as tumor associated macrophages (TAMs), Tregs, and dendritic cells (DCs), within the tumor microenvironment (TME)suppresses effector T cells. The role of NF-κB in driving the phenotypes of these immune cells and the impact of targeting the canonical or non-canonical NF-κB pathways in PDAC is largely unclear and should be investigated. Until then, it is important to appreciate the role of the NF-κB pathway in the development of each immune cell type. Both the canonical and non-canonical pathways are essential for normal differentiation and self-renewal of hematopoietic stem cells [53,54]. Vav-Cre driven deletion of RELA, which ablates RELA expression in all hematopoietic cells, resulted in accumulation of hematopoietic stem cells that are defective in further differentiation into progenitors [53]. Interestingly, myeloid-specific deletion or pharmacologic suppression of IKKβ resulted in granulocytosis and rendered mice more susceptible to endotoxin-induced shock due to increased circulating IL-1β and TNFα [55]. On the other hand, bone marrow transplant experiments showed that IKKβ-deleted stem cells failed to mature into T cells due to overwhelming TNFα-induced apoptosis, and this defect could be fixed/prevented by co-deletion of TNF receptor (TNFR) [56]. Tightly regulated canonical NF-κB activity is essential for positive and negative selection of major histocompatibility complex (MHC)-1 restricted CD8^+^ T cell selection, as these processes are abrogated by excessive or inadequate canonical NF-κB activity mimicked by expression of activated IKKβ mutant or dominant negative IκB in T cells [57]. Intriguingly, these mutant T cells retained a normal ability to undergo MHC-II restricted CD4^+^ T cell selection, suggesting that the canonical NF-κB activity is dispensable in CD4^+^ T cell selection [57]. That said, RELA is critical for maintenance of tolerogenic CD4^+^ Foxp3^+^ Treg as deletion of RELA in this subset induces autoimmune disorders [58]. The activation of NF-kB downstream of MyD88 has a critical role in the activation and functionality of MDSCs. The ability of MyD88^−/−^ MDSCs to suppress the activity of T cells and secrete immunoregulatory cytokines was considerably reduced compared to the wild-type MDSCs both in vitro and in vivo. Also, the activation of NF-κB signaling in TAMs contributes to carcinogenesis in various models of inflammation-associated cancers including PDAC [59]. In B cells, IKKβ is essential for survival, proliferation, maturation, and mounting antibody response to T cell dependent and independent antigens [60,61,62].

To date, immunotherapy, specifically "immune checkpoint inhibitors" (ICIs) and chimeric antigen receptor (CAR) T cells, remains largely unsuccessful in PDAC. Attempts to relieve T cell checkpoints with anti- (programmed death) PD-1/anti-PD- ligand(L)1 and/or anti- cytotoxic T-lymphocyte-associated protein 4 (CTLA4) are inadequate in mounting an effective therapeutic response. One of the major obstacles is T cell exhaustion, which is driven by upregulation of the transcription factors nuclear factor of activated T cells (NFAT), basic leucine zipper ATF-like transcription factor (BATF), and interferon regulatory factor 4 (IRF4) [63,64,65]. However, the molecular mechanisms that upregulate these factors remain largely unclear. Chronic engagement of the Toll-like receptor (TLR)7 or infection with HIV leads to anergy of CD4^+^ T cells via NFAT cytoplasmic 2 (NFATc2) [66]. Sustained engagement of T cell receptors or TLR engagement, as expected within the inflammatory TME of PDAC, may contribute to the upregulation of these exhaustion factors, but this speculation remains to be tested.

## 4. Mechanisms that Activate the NF-κB Pathway in PDAC

**Figure 1:**
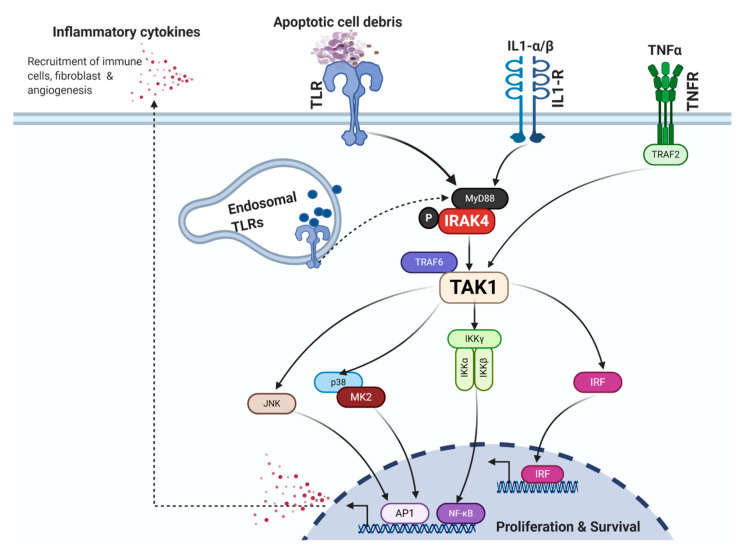
Overview of receptors and signaling pathways that activate the NF-κB cascade. After recognition of their cognate ligands, IL1-R and TLR and recruit their adaptor protein, MyD88. MyD88 then associates with IRAK4 recruiting TNFR- receptor-associated factor (TRAF)6, transforming growth factor-β (TGF-β)-activated kinase 1 (TAK1), IKK complex, and activating NF-κB, JNK and p38 MAPKs, and type-1 interferon pathways. In addition, engagement of the TNFRs recruits TNFR receptor-associated factor (TRAF)2, activating TAK1.

The Toll-like/Interleukin-1 receptor (TIR) and tumor necrosis factor receptor (TNFR) family members are the main triggers that drive the canonical NF-κB pathway in PDAC cells and CAFs. The TLR1-10 in humans specialize in sensing both damage-associated molecular patterns (DAMPs) and pathogen-associated molecular patterns (PAMPs) and are sentinels that initiate inflammation as part of the innate immune response. Engagement of TLRs results in cytoplasmic aggregation of adaptor proteins, including myeloid differentiation factor-88 (MyD88), TIR domain-containing adapter-inducing interferon-β (TRIF), TRIF-related adaptor molecule (TRAM), and sterile-α and armadillo motif-containing proteins [67]. Specifically, MyD88 oligomerizes with the closely homologous IRAKs, including IRAK1, IRAK2, and IRAK4, whereby IRAK4 phosphorylates IRAK1, leading to recruitment of TNFR receptor-associated factor (TRAF)6, transforming growth factor-β (TGF-β)-activated kinase 1 (TAK1), IKK complex, and activation of the NF-κB, p38/mitogen-activated protein kinases (MAPK) and type-1 interferon pathways [68]. Additionally, engagement of the TNFRs leads to recruitment of TRAF2, which polyubiquitinates and activates receptor-interacting protein kinase (RIPK), which in turn binds and activates TAK1 [69,70] (Figure 1). In the following sections, we will review the role of these pathways in PDAC.

### 4.1. Toll-Like Receptors 

TLRs are ubiquitously expressed type I transmembrane receptors consisting of an extracellular domain, transmembrane region, and intracellular domain [71]. The extracellular domain contains leucine-rich repeats and cysteine residues which identify and bind to evolutionary conserved regions in DAMPs or PAMPs, whereas the intracellular domain is greatly homologous amongst TLRs containing the TIR domain which is important for the intracellular activation of signaling cascades resulting in the secretion of cytokines and chemokines [72]. The role of TLRs in PDAC pathophysiology is highly context dependent and cell type-specific. In this review, we will focus mainly on TLR4, TLR7, and TLR9, for which more literature relevant to PDAC are available. 

#### 4.1.1. TLR4

Enhanced expression of TLR4 is found in neoplastic, stromal and inflammatory cells in PDAC [73]. Treatment with lipopolysaccharide (LPS), a ligand for TLR4, promotes the invasiveness of PDAC cells through enhanced production of matrix metallopeptidase 9 (MMP9) [74]. In the genetic p48-Cre:KRAS^G12D^ mouse model, LPS treatment accelerated stepwise progression from precancerous lesions to PDAC, which could be blocked by inhibiting TRIF, one of the two major downstream adaptor proteins recruited by TLR4. On the other hand, blockade of MyD88, the other adaptor protein downstream of TLR4, paradoxically exacerbated stromal inflammation and accelerated PDAC development through expansion of dendritic cells, which promotes development of antigen-specific Th2-deviated CD4^+^ T cells. Importantly, the pro-tumorigenic effect of LPS was lost when p48-Cre:KRAS^G12D^ was transplanted with Tlr4^−/−^ bone marrow, demonstrating the critical role of TLR4 agonism in immune cells in inflammation-induced PDAC progression [73].

#### 4.1.2. TLR7/8

Expression of TLR7 is upregulated in neoplastic ductal and inflammatory cells in PDAC [75]. Treatment of p48-Cre:KRAS^G12D^ mice with a TLR7 agonist greatly accelerated stromal expansion and tumor progression. The tumor stimulating effect of TLR7 was mediated by the downregulation of p16, cyclin D1, and PTEN, and the upregulation of p53, p27, p21, c-Myc, cyclin B1, SHPTP1, PPARγ, and TGF-β. Conversely, the TLR7 inhibitor protected p48-Cre:KRAS^G12D^ mice from caerulein-induced PDAC progression through downregulation of p21, p27, p-p27, cyclin B1, CDK4, and p-STAT3. Notably, caerulein-induced PDAC progression was completely blocked in p48-Cre:KRAS^G12D^ mice transplanted with TLR7^−/−^ bone marrow, suggesting that TLR7 in inflammatory cells is essential in PDAC progression [75]. Another report also showed upregulated expression of TLR7 and TLR8 in neoplastic ductal cells from human PDAC samples compared to normal pancreas or pancreatitis tissues [76]. PANC-1 cells overexpressing TLR7/TLR8 had higher NF-κB activity and COX-2 expression and exhibited decreased chemosensitivity. Overall, these studies support inhibiting TLR7/8 in PDAC as a therapeutic strategy.

On the other hand, other studies support activating TLR7/8 as a therapeutic strategy, mainly through promoting an immune-mediated anti-tumor response. In a syngeneic orthotopic mouse model, TLR7/8 agonist 3M-011 stimulated antigen presentation by dendritic cells following local radiotherapy, thereby boosting systemic and local immune-mediated tumor rejection [77]. Similarly, an impressive systemic anti-tumor immune response was elicited by irreversible electroporation (IRE) of local PDAC tumors that were co-treated with an intratumoral TLR7 agonist (1V270) and systemic anti-PD-1 receptor checkpoint blockade. This combination resulted in an abscopal effect that eradicated untreated distant tumors [78]. Another study with syngeneic orthotopic models showed that TLR7/8 agonist R848 treatment resulted in increased intratumoral infiltration of CD8^+^ and CD4^+^ T cells and reduced Treg frequency. In addition, mice treated with R848 showed improvements in molecular and behavioral cachexia manifestations. These changes led to doubling of survival time. Importantly, R848 paradoxically promoted neoplastic growth of tumors grown in TLR7^−/−^ mice, suggesting that the beneficiary effects of TLR7 agonism are mediated entirely through host cells, including the immune system and stromal cells [79]. However, the detailed molecular mechanisms by which TLR7/8 support antigen presentation by dendritic cells and promote anti-tumor T cell response are largely unclear.

#### 4.1.3. TLR9

Expression of TLR9 increases in epithelial, stromal, and immune cells during PDAC progression [80]. Treatment with a TLR9 ligand accelerated neoplastic progression in p48-Cre:KRAS^G12D^ mice through enhanced secretion of pro-inflammatory cytokines, including chemokine (C-C motif) ligand (CCL)11, from the neoplastic cells. These cytokines not only propel pancreatic stellate cells into an inflammatory phenotype, but also draw an influx of immunosuppressive myeloid cells and Tregs [80]. In addition, expression of TLR9 in PDAC cells can be further induced following DNA damage caused by chemotherapy, particularly irinotecan, leading to activation of IRAK4 and TPL2 kinases and the downstream NF-κB and MAPK pathways. These events sustain cellular survival to allow DNA damage repair [81]. In addition, TLR9 stimulation promotes vascular endothelial growth factor (VEGF) and platelet-derived growth factor (PDGF) production, as well as expression of the anti-apoptotic protein Bcl-xL [82]. On the other hand, TLR9 agonism by immunomodulatory oligonucleotides cooperates with cetuximab in curbing orthotopic growth of an AsPc-1 xenograft [83]. Synthetic TLR9 agonists (CpG-ODNs) are oligodeoxynucleotides which contain CpG motifs and have been widely used as anti-allergic agents or vaccine adjuvants [84]. In an orthotopic model using human GER carcinoma cell line, mice treated with CpG-ODNs had reduced metastases in the diaphragm, liver and spleen [85]. Moreover, the combination of CpG-ODNs and gemcitabine caused a delay in the development of bulky disease (extensive peritoneal tumor burden), decreased metastasis, and enhanced survival time in comparison to gemcitabine treatment alone. Furthermore, TLR9 agonists have been shown to have therapeutic value, mainly through stimulating an anti-tumor response. In a Panc02 model expressing ovalbumin, the combination of a vaccine based on immune stimulatory complexes (ISCOM) and a TLR9 agonist could restore anti-tumor immune response by activating NK cells, cytotoxic T cells, and dendritic cells, leading to tumor regression [86]. The context dependent role of TLR9 in PDAC progression and treatment resistance underscores the importance of careful consideration of therapeutic strategies towards TLR9.

### 4.2. IL-1α/β and IL-1R

Enhanced systemic and intratumoral expression of IL-1α and IL-1β is common in PDAC patients. In GEMM, expression of oncogenic KRAS drives the IKKβ-NF-κB axis via autocrine expression of IL-1α [87]. Activated NF-κB further increases expression of the target gene p62, which promotes ubiquitination of TRAF6 which feeds back to the canonical NF-κB cascade. This process is critical for PDAC development. Secretion of IL-1β by tumor cells and stromal CAFs leads to increased NF-κB activity in both cell types, increased intratumoral collagen deposition and chemoresistance [49]. These studies provide a solid rationale for targeting IL-1R in combination with chemotherapy in PDAC. Recently, tumor-derived IL-1β was shown to foster an immunosuppressive TME by promoting M2 macrophage polarization and an influx of myeloid-suppressor cells, such as regulatory B and Th17 cells. On this basis, neutralizing the IL-1β antibody promotes intratumoral CD8^+^ T cell infiltration and synergizes with anti-PD1 [88].

### 4.3. TNF-α and TNFR

The PDAC TME is rife with TNFα secreted by PDAC cells and also immune cells, such as macrophages [89,90]. TNF-α and Regulated upon Activation, Normal T Cell Expressed and Presumably Secreted (RANTES) secreted by macrophages drive NF-κB activity in PDAC cells, resulting in the upregulation of several target genes, including AKT1, Bcl-xL, Bcl2A1, COX-2, CDK1, CCND1, PDGFβ, and several genes encoding matrix metallopeptidases. These genes result in acinar to ductal metaplasia (ADM) and progression to pancreatic intraepithelial neoplasia (PanIN) [89]. High intratumoral TNFα expression is associated with poor prognosis in PDAC patients [91]. The addition of anti-TNFα neutralizing antibodies infliximab or etanercept reduced tumor desmoplasia and cooperated with chemotherapy in delaying tumor growth and mouse survival [91]. In human xenograft mouse models, infliximab reduced AP-1 and NF-κB activity and attenuated PDAC growth and metastasis [90]. Unfortunately, the addition of etanercept failed to improve treatment response with gemcitabine in a phase 1/2 clinical trial [92]. It is becoming clear that TNFR signaling can trigger both pro-apoptotic and anti-apoptotic pathways. Therefore, it is critical to further dissect the contribution of downstream signaling cascades in order to devise therapeutic strategies that are more likely to be successful in the clinic. 

### 4.4. IRAK4 

IRAKs, which consist of four family members (IRAK1–4), are the key signal transducers for IL-1R and TLRs [93]. Activation of the TIR family member receptors results in recruitment of the adaptor protein MyD88, the IRAKs and TRAF-6. IRAK4 undergoes autophosphorylation at several residues, including Thr^209^ and Thr^387^, and subsequently phosphorylates IRAK1, resulting in the dissociation of IRAK1 and TRAF6 from the active complex [94]. After dissociation, IRAK1 binds to TAB-1, TAB-2, and TAK-1 (transforming growth factor-β-activated kinase), leading to activation of TAK-1 which phosphorylates the IKK complex (IKKα, IKKβ, and IKKγ), JNK, and the p38 MAPKs [95].

In PDAC, the kinase activity of IRAK4, but not IRAK1, is essential for downstream signal transduction [36], making it an actionable target. In the absence of stimulation by a TLR ligand, constitutive phosphorylation of IRAK4 was detected in 11 out of 12 human PDAC cell lines but not in the non-transformed pancreatic ductal cell lines, human pancreatic nestin expressing (HPNE) and human pancreatic duct epithelial (HPDE) cell lines. Immunohistochemical (IHC) staining of p-IRAK4 was evident in approximately 60% of human PDAC samples and strongly correlated with p-RELA staining. The presence of p-IRAK4 was predictive of higher postoperative relapse and poorer patient survival. Suppression of IRAK4 strongly decreased NF-κB activity, chemoresistance, anchorage independent growth, and production of several pro-inflammatory cytokines, including IL-1β, IL-6, IL-8, CXCL1, CXCL2, and CCL2. As cytokines/chemokines have been shown to induce desmoplasia [96], IRAK4 inhibition led to the impairment of the ability of PDAC cells to stimulate proliferation, invasion, and migration of CAFs. Notably, strong p-IRAK4 and p-RELA IHC staining is also present in stromal CAFs. Targeting IRAK4 markedly reduced NF-κB activity and production of collagen and IL-1β by CAFs [49].

IRAK4 is critical in innate immunity against microorganisms. IRAK4^−/−^ mice are immunocompromised and do not respond to challenges by TLR ligands [97]. IRAK4-deficient patients are susceptible to invasive bacterial infections in infancy and early childhood [98,99]. However, the role of IRAK4 in immune cells in PDAC development and immune evasion has not been investigated. Because TLR7 and TLR9 signal exclusively through IRAK4 and contribute to inflammation-associated PDAC development, it is foreseeable that IRAK4 in immune cells also contributes to PDAC development. While IRAK4 is critical for a TLR-mediated response, its role in T cell receptor-mediated responses remains controversial. Using almost identical in vitro and in vivo stimulation assays but independently generated IRAK4^−/−^ C57BL/6 mice, Suzuki et al showed that IRAK4 is absolutely essential for T cell activation [100], whereas Kawagoe et al showed that IRAK4 is dispensable [101]. In the context of anti-tumor T cell response, it is critical to carefully evaluate the role of IRAK4 in initial MHC-restricted T cell activation. On the other hand, because chronic T cell receptor engagement is a potential mechanism that drives T cell exhaustion [102], a universal phenomenon in PDAC, targeting IRAK4 may be a strategy to revitalize anti-tumor T cells. Therefore, the utilization of IRAK4 inhibition in immune-oncologic regimens must be carefully evaluated preclinically.

### 4.5. TAK1 

Transforming growth factor-β (TGF-β)-activated kinase 1 (TAK1) is a serine/threonine kinase in the family of mitogen-activated protein kinase (MAP3K) [103]. It is also known as MAP3K7. TAK1 has a critical role in inflammation and cell survival by serving as the signaling hub downstream of several receptors, including IL-1R, TLR, TGFβ, and TNFRs, and upstream of the JNK, MAPK, NF-κB, and activator protein-1 (AP-1) pathways [104,105]. Upon receptor engagement, TAK1 undergoes K63-linked polyubiquitination, specifically at the K158 residue, by E2 ligase UBC13/UEV1A and E3 ligase TRAF2/TRAF6 [106,107]. Once polyubiquitinated, TAK1 undergoes autophosphorylation at T184, T187 and S192 to become fully activated [108,109,110]. On the other hand, TAK1 is negatively regulated by several mechanisms. For instance, de-ubiquitinating enzymes, including ubiquitin-specific peptidases-4 (USP4) and CYLD, remove K63-polyubiquitination of TAK1, thereby blocking its activation [111,112,113]. Furthermore, Itch E3 ubiquitin ligase mediates the K48-linked polyubiquitination of TAK1 at K72 and targets it for degradation [113]. In addition, TAK1 is dephosphorylated by phosphatases including protein phosphatase 6, protein phosphatases 2C family members, and dual-specificity phosphatase 14 [114,115,116].

Global deletion of TAK1 in mice results in profound vascular maldevelopment and early embryonic lethality [117,118]. Tissue-specific deletion of TAK1 in enterocytes results in rapid development of intestinal inflammation driven by IL-1β, TNFα, and MIP2, followed by massive apoptosis of enterocytes, all of which are attenuated in TNFR1-deleted mice [119]. Therefore, TAK1 is critical in maintaining the homeostasis of intestinal epithelial cells by driving survival genes to evade the pro-apoptotic effect of TNFα. In colon cancer cells, oncogenic KRAS stimulates bone morphogenetic protein (BMP)-7 secretion and autocrine BMP signaling, leading to TAK1 activation and activation of the Wnt/β−catenin pathway [120]. Whether similar signaling exists in PDAC remains to be investigated. Targeted deletion of TAK1 in the pancreas has not been published. However, TAK1 is critical in maintaining cellular survival following genotoxic stress in PDAC. DNA damage results in cytoplasmic translocation of ataxia telangiectasia mutated (ATM), which activates TRAF6 and, subsequently, TAK1 and the downstream IKK complex [121,122]. In addition, TAK1 can be phospho-activated at S412 by p21 activated kinase 1 (PAK1), whose activity is elevated by docking with gemcitabine [123]. Accordingly, RNAi-mediated silencing or pharmacologic inhibition of TAK1 using LYTAK1 potentiate the cytotoxic effect of various chemotherapeutics, including gemcitabine, in preclinical PDAC models [124].

Because TAK1 functions as the signaling hub downstream of several immune receptors, it is critical to carefully appraise the impact of targeting TAK1, especially when combined with immunosuppressive chemotherapies, or development of immunotherapy. To this end, several elegant studies have provided important insights. Mxl-Cre driven deletion of TAK1 in hematopoietic cells and hepatocytes leads to rapid apoptosis of hematopoietic cells and hepatocytes, resulting in pancytopenia and liver failure [125]. The essential role of TAK1 in T cell development and maturation is rather well-characterized. Lck-Cre driven deletion of TAK1 leads to impaired thymocyte development and a marked decrease in T cells in peripheral tissues. Upon stimulation with anti-CD3e, TAK1-deleted T cells are impaired in NF-κB and JNK activation and are more prone to apoptosis [126]. However, TAK1-deleted T effector cells retain normal NF-κB activation and cytokine production upon T cell receptor activation, although they are impaired in activating p38 in response to IL-2, -7, and -15 [127]. Similarly, TAK1-deleted B-cells are defective in response to TLR, CD40, and B cell receptor stimulation [128]. Mice with B-cell-specific TAK1 deletion have impaired B cell development and maturation and are defective in mounting antigen-specific antibody responses [129]. Intriguingly, myeloid-specific deletion of TAK1 results in neutrophilia and myeloproliferative disorder. TAK1-deleted neutrophils exhibit enhanced activation of NF-κB, p38, and JNK upon LPS treatment. Mechanistically, TAK1-deleted myeloid cells showed higher baseline TAB1-p38 interaction, potentially raising the baseline activity of p38 and priming p38 to a higher activated state upon LPS stimulation [130]. Overall, these genetic-based studies are invaluable in elucidating the cell-type specific role of TAK1. From the therapeutic perspective, the paradoxical increase in the number and activity of myeloid cells following TAK1 deletion provided some confidence that TAK1 inhibition may not aggravate neutropenia that is commonly associated with chemotherapy. However, the potential adverse impact on reticulocytes and megakaryocytes should be cautioned. In addition, the essential role of TAK1 in T and B cell development and maturation should also be considered when designing immunotherapy-based approaches in PDAC. To this end, the recent advent of TAK1 inhibitors, LYTAK1 and Takinib, provides an opportunity to evaluate the immunotherapeutic value of targeting TAK1 in PDAC [124,131].

### 4.6. TPL2

Tumor progression locus 2 (TPL2, also known as MAP3K8 or COT) is a serine/threonine protein kinase that mediates TLR, IL-1, and TNF receptor dependent MAPK and NF-κB activation [132,133]. TPL2 mRNA consists of an internal start codon, giving rise to two TPL2 protein isoforms of 58kDa and 52kDa. In the absence of receptor stimulation, TPL2 is bound to NF-κB1/p105 protein complexed with the A20-binding inhibitor of NF-κB (ABIN)-2. This binding keeps TPL2 inactive and stable. LPS, IL-1β, or TNFα stimulation results in activation of IKKβ which phosphorylates p105, resulting in its degradation to p50 and the release of the TPL2 protein [134,135]. TPL2 also undergoes phosphorylation at Ser400 by IKKβ and at Thr290 by an unknown kinase to become fully activated [136,137,138]. Activated TPL2 phosphorylates MEK1/2 and p105 which cause ERK1/2 and p50 NF-κB transcription factor activation, respectively [139,140]. In addition, TPL2 has also been shown to phosphorylate RELA/p65 NF-κB subunit at its Ser276 residue, MKK4/SEK1 (proximal kinase of JNK) and MKK3/6 (proximal kinase of p38α) in fibroblasts, and macrophages stimulated with TNF-α or LPS [133,141,142]. In PDAC cells, TPL2 is activated via a KRAS-MAPK driven IL-1β autocrine signaling loop [81]. In this setting, inhibition of TPL2 suppresses both MAPK and NF-κB pathways. When exposed to genotoxic stress, TLR9 is upregulated, which engages IRAK4 and TPL2 to amplify both MAPK and NF-κB pathways. This study clearly establishes TPL2 as a novel therapeutic target for PDAC. In other cancer types, such as melanoma and ovarian cancer, TPL2 becomes oncogenic by overexpression, or acquires gain-of-function truncations, fusions, and point mutations, leading to hyperactive MAPK, NF-κB, JNK, and p38 cascades. To date, clinical grade TPL2 inhibitors remain unavailable and should be developed.

## 5. Intricate Crosstalk between the KRAS and NF-κB Pathways

Oncogenic KRAS mutations occur in >90% of PDAC [143] and KRAS itself is a major driver of NF-κB activity. Downstream of the KRAS oncoprotein, the PI3K-AKT-mTOR effector promotes phosphorylation of IKK, leading to increased nuclear translocation of RELA [144,145,146]. The RalGDS-RALB axis binds to Sec5 to activate TBK1, leading to activation of noncanonical IKKε [147]. Furthermore, the KRAS oncoprotein was shown to transcriptionally upregulate GSK-3α and GSK-3β, which stabilizes the TAK1–TAB1 complex, resulting in the constitutive activation of the canonical NF-κB signaling cascade [148].

**Figure 2:**
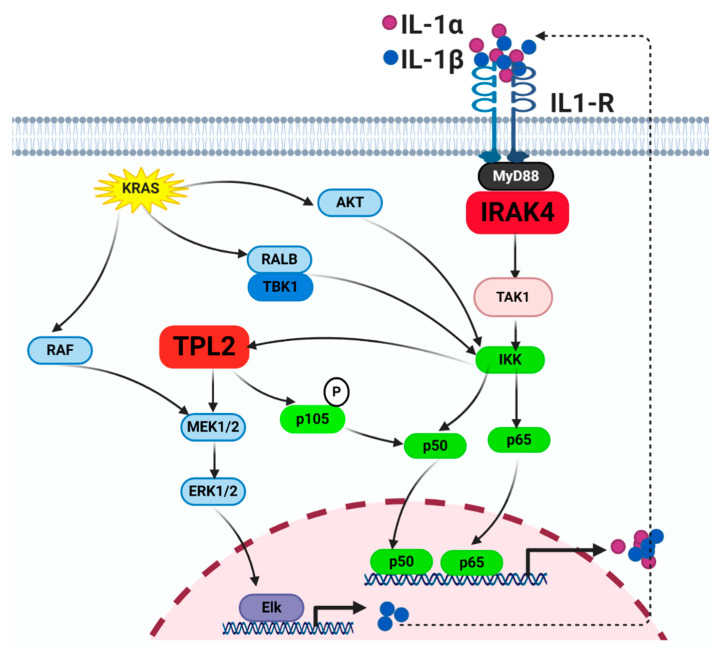
Signaling crosstalk between the KRAS and NF-κB pathways. The crosstalk of oncogenic KRAS with NF-κB pathway to promote cellular proliferation, survival, and secretion of cytokines.

Contribution of the RAF-MEK-ERK cascade to the NF-κB cascade is indirect and mediated through autocrine IL-1β production. Through the RAF-MEK-ERK cascade, the KRAS oncoprotein markedly upregulates production of IL-1β, which, in an autocrine manner, engages the IL-1R-IRAK4-TPL2 axis to activate the canonical NF-κB pathway and further reinforces MEK-ERK activity [81]. Importantly, ablation of IRAK4 completely blocks KRAS-induced transformation and tumorigenesis [87]. In PDAC GEMM, oncogenic KRAS-driven progression to PDAC absolutely requires the autocrine IL-1α-IKKβ- NF-κB axis [87], as deletion of IKKβ completely abrogated PDAC development in these mice. In support, ablation of IRAK4 completely abrogated RAS-induced transformation. Furthermore, NF-κB activation enhanced the activated level of KRAS mutant proteins as assayed by RAS-GTP levels, leading to accelerated PDAC development [149]. These studies widen the spectrum of oncogenic RAS signaling beyond the direct effectors and include inflammation as an equally critical component (Figure 2). Because targeting KRAS and its direct effectors, including the RAF-MEK-ERK and PI3K-AKT-mTOR cascades, remains largely unsuccessful, we propose that targeting key signaling nodes in the canonical NF-κB cascades could represent another promising anti-RAS strategy.

## 6. Therapeutic Targeting of the NF-ĸB Pathway in PDAC 

Several hundreds of agents have been proposed to have anti-NF-κB activities [150]. On the one hand, this scenario highlights the importance of this pathway in cancer therapy. On the other hand, it accentuates the lack of specific inhibitors that can effectively and safely curb this pathway in the clinic. Small peptides or peptidomimetics that directly interfere with NF-κB dimerization or binding with DNA have been published in preclinical settings [151,152,153], but these have not been advanced into clinical trials. Therefore, much attention is paid towards targeting the signaling nodes, especially kinases, that activate NF-κB. In solid malignancies, including PDAC, the canonical pathway is the predominant mechanism that drives NF-κB and is triggered by inflammatory cytokines [31].

Despite the plethora of preclinical studies employing various “NF-κB targeting” agents, only a few of these candidates have actually entered and completed early phase clinical trials. Although the COX2 inhibitor celecoxib was shown to abrogate NF-κB activity and cooperate with gemcitabine in preclinical studies [149,154], the addition of 400 mg celecoxib twice daily and 81 mg aspirin once daily did not improve the therapeutic efficacy of gemcitabine in a phase II clinical trial [155]. In phase II studies, curcumin given at 8 g/day alone or in combination with gemcitabine showed preliminary biological activity in a few selected PDAC patients [156,157], but it is unclear whether larger clinical trials are being planned. Blocking the degradation of IκB with the proteasome inhibitor bortezomib, which also affects numerous other substrates, did not potentiate gemcitabine in a phase II clinical trial [158]. Despite these setbacks, the recent better understanding of the signaling mechanisms that drive NF-κB activity in PDAC has opened up more opportunities. Targeting IL-1R and IRAK4 is more promising, as active agents are now available or being tested in clinical trials. Recently, dendritic cell vaccination has emerged as a novel strategy to prime host anti-tumor immunity [159]. Specifically, the combination of a dendritic cell vaccine with gemcitabine led to eradication of orthotopic tumors and provided durable protection against PDAC in mouse models [160]. However, whether the NF-κB cascade is involved in antigen presentation by dendritic cells and priming of T cells remains unclear and warrants further investigation. At present, no IKK, TPL2, or TAK1 inhibitors are available for further testing in clinical trials for PDAC.

### 6.1. IL-1R Blockade

Because autocrine IL-1R signaling is a critical component that drives the canonical NF-kb cascade in PDAC, the combination of the IL-1R antagonist Anakinra with nab-paclitaxel, gemcitabine, and cisplatin has been opened in a pilot clinical trial for patients with resectable or potentially resectable PDAC (NCT02550327). In addition, canakinumab (a humanized neutralizing IL-1b antibody) and rilonacept (an IL-1 TRAP) are available for further testing. Canakinumab is currently FDA-approved for treatment of adult onset Still’s disease. 

### 6.2. IRAK4

CA-4948 is an orally available, specific IRAK4 inhibitor that is now being tested as a single agent for patients with relapsed/refractory hematologic malignancies. Interim results showed CA-4948 at 200 mg twice daily to be generally well tolerated and showing preliminary efficacy [161] (NCT03328078). The combination of IRAK4 inhibitors with chemotherapy is supported by preclinical studies [49,81,162] and should be advanced into clinical trials.

## 7. Conclusions and Perspectives

Chronic inflammation, driven by the NF-κB pathway, has a major role in every aspect of PDAC pathobiology, ranging from initiation, progression, and metastasis to treatment resistance. In addition, due to the essential role of this pathway in KRAS-induced PDAC progression, the NF-κB pathway has been, and will undoubtedly remain, an attractive therapeutic target. However, targeting the NF-κB factors and the immediate upstream IKK has been challenging due to the lack of specific and clinically safe therapeutic agents, likely due to the essential role of these targets in normal physiology. With recent understanding of the upstream mechanisms that drive NF-κB in PDAC, novel therapeutic targets have begun to surface. Aside from combination with chemotherapy, targeting the NF-κB pathway as a strategy to potentiate immunotherapy has begun to draw attention. As immunotherapy is not without side effects, it is imperative to gain a deeper and more comprehensive understanding of the role of NF-κB pathway in each cellular compartment, and even in different immune subsets, prior to advancing any therapeutic combinations into clinical trials. In particular, these studies should be conducted in clinically-relevant settings, such as in GEMMs of humanized mouse models, in which the net impact of systemic NF-κB targeting agents can be assessed. In summary, targeting inflammation through the NF-κB pathway remains a valid direction and warrants more intensive and concerted investigation from the research community.
